# Treatment of Aniline Wastewater by Membrane Distillation and Crystallization

**DOI:** 10.3390/membranes13060561

**Published:** 2023-05-30

**Authors:** Fangli Zhang, Wei Hou, Zhonglin Yang, Zhaohui Wang, Rizhi Chen, Enrico Drioli, Xiaozu Wang, Zhaoliang Cui

**Affiliations:** 1 State Key Laboratory of Materials-Oriented Chemical Engineering, College of Chemical Engineering, Nanjing Tech University, Nanjing 210009, China; 18437902868@163.com (F.Z.); 202161204426@njtech.edu.cn (W.H.); zhwang@njut.edu.cn (Z.W.); rizhichen@njtech.edu.cn (R.C.); 2 National Engineering Research Center for Special Separation Membrane, Nanjing Tech University, Nanjing 210009, China; 3 SINOPEC Nanjing Research Institute of Chemical Industry Co., Ltd., Nanjing 210048, China; yangzhonglin07@163.com; 4 Jiangsu National Synergetic Innovation Center for Advanced Materials (SICAM), Nanjing Tech University, Nanjing 210009, China; 5 Research Institute on Membrane Technology, ITM-CNR, Via Pietro Bucci 17/C, 87036 Rende, Italy; e.drioli@itm.cnr.it

**Keywords:** membrane distillation, membrane crystallization, Fenton oxidation, aniline wastewater

## Abstract

Aniline is a highly toxic organic pollutant with “carcinogenic, teratogenic and mutagenesis” characteristics. In the present paper, a membrane distillation and crystallization (MDCr) process was proposed to achieve zero liquid discharge (ZLD) of aniline wastewater. Hydrophobic polyvinylidene fluoride (PVDF) membranes were used in the membrane distillation (MD) process. The effects of the feed solution temperature and flow rate on the MD performance were investigated. The results showed that the flux of the MD process was up to 20 L·m^−2^·h^−1^ and the salt rejection was above 99% under the feeding condition of 60 °C and 500 mL/min. The effect of Fenton oxidation pretreatment on the removal rate of aniline in aniline wastewater was also investigated, and the possibility of realizing the ZLD of aniline wastewater in the MDCr process was verified.

## 1. Introduction

Aniline is a kind of highly toxic, refractory organic pollutant with “carcinogenic, teratogen and mutagenic” properties, and is among the 129 priority environmental pollutants in the United States and is included in the pollution blacklist published by the European Community [1,2]. Furthermore, about 30,000 t aniline is discharged into the environment every year worldwide [3,4,5,6]. As of 2019, the domestic aniline production capacity is more than 3.6 million t/a. Aniline wastewater is characterized by a high content of aniline substances (mass concentrations can reach thousands of mg/L [5]), a high salt content, obvious changes in the chemical oxygen demand (COD) content (ranging from 200 mg/L to 2000 mg/L [7]), etc. Aniline wastewater is mainly derived from the production and use process and widely exists in the printing and dyeing, pharmaceutical, and papermaking industries [8,9]. The treatment of aniline wastewater traditionally involves chemical, physical, biological, and electrochemical methods [10]. Among them, the biological method is the most widely used, through which aniline can be made completely biodegradable to CO_2_ and N_2_/NOx [11]. However, chemical, biological, and electrochemical methods cannot realize the recovery of aniline and discharge a large number of chemical reagents, which not only increases the operating cost but also causes secondary pollution. For example, biological methods are temperature-dependent and are restrained by the toxicity of pollution, especially because they are very sensitive to the concentration of salt in wastewater. The electrochemical method is clean and easy to handle, but it produces more carbon dioxide, which is called greenhouse gas. Aniline wastewater can also be treated with biological enzymes such as Laccases [12], but the interaction mechanism of biological enzymes with different types of wastewater is still unclear and it has relatively high costs. Although aniline can be recovered by physical methods, they are not suitable for industrial applications because it is difficult to separate the extractant and aniline [13]. Therefore, an efficient aniline wastewater treatment process is urgently needed; it is necessary to develop a new process for the treatment of aniline wastewater.

As a new desalination technology, membrane distillation (MD) is especially suitable for treating high-salt solutions [14,15]. In the MD process, a hydrophobic porous membrane acts as the physical barrier between the hot feed and the permeate flow, and the vapor pressure gradient drives the volatile compounds to be transported from the feed side to the permeate side [16]. Since the need for transmembrane pressure is eliminated in MD, this operation is not sensitive to feed concentration [17]. In addition, MD operates at a low temperature below the boiling point of the feed solution, generating water vapor and utilizing renewable heat energy [18]. There are four different modes of MD: Direct Contact Membrane Distillation (DCMD) [19,20]; Air Gap Membrane Distillation (AGMD) [21,22]; Sweeping Gas Membrane Distillation (SGMD) [23,24]; and Vacuum Membrane Distillation (VMD) [25,26]. Compared with the other MD configurations, VMD permits higher partial pressure gradients, and, hence, higher permeate flux can be achieved [27,28]. In addition, it is better than DCMD in terms of energy consumption/permeate flow ratios and thermal evaporation efficiency [29]. One of the most important benefits provided by VMD is that of the possibility of harnessing available renewable energy sources such as solar energy [30,31]. In this study, the VMD configuration was also adopted to carry out membrane distillation and crystallization (MDCr) experiments [32]. In addition, in the treatment of specific wastewater, the unique characteristics of MD allow only volatile components to pass through the pores, resulting in the simultaneous generation of water and recyclable materials [33]. It is also noteworthy that the process can concentrate brine into a supersaturated state for further MDCr processes [34]. Before the MDCr process, the salinity of feed brine can be concentrated to be close to the saturation state by MD. Thus, MDCr can obtain clean water and salt crystals in one step, and, with some additional and simple treatments, can even meet drinking water standards. The nucleation and growth of salt crystals can be controlled by adjusting the feed temperature and flow rate [35]. In addition, the MDCr process can start with a low-level heat source, such as waste heat and solar energy from a factory or power plant [36,37]. Therefore, it is feasible to integrate the MDCr process into the zero liquid discharge (ZLD) system [38]. In sewage treatment, the concept of ZLD is mentioned more and more frequently; ZLD is a concept where most industrial raw materials, such as water and salts, are purified, reused, and separated after multiple stage cycles as much as possible to ensure that no waste liquid is discharged from a factory [39,40].

In this work, the VMD process was used to investigate the treatment feasibility of aniline wastewater. The temperature and flow rate during VMD operation was optimized, and the contamination conditions under different conditions were investigated. The Fenton oxidation process was used to remove aniline from wastewater, and the optimal concentration of H_2_O_2_ and FeSO_4_ was determined. The pretreatment technology of aniline wastewater was also studied for a better treatment effect. Then, a long-time MDCr experiment was carried out with aniline wastewater, and regular cube salt crystal was obtained, showing the feasibility of membrane distillation crystallization to treat aniline wastewater.

## 2. Experiment

### 2.1. Material

Commercial hydrophobic flat sheet PVDF (GVHP 04700, 0.22 μm, Millipore, Burlington, MA, USA) membranes were applied in this work. Ferrous sulfate (FeSO_4_) and hydrogen peroxide (H_2_O_2_) are the materials for the Fenton reaction. Aniline wastewater came from the production of anti-aging agents by the Nanjing Research Institute of Chemical Industry. Both FeSO_4_ and H_2_O_2_ came from the China National Pharmaceutical Group, with analytical purity and an H_2_O_2_ concentration of 30%. Ionized water was produced by a self-made reverse osmosis system. All chemicals were used without further purification.

### 2.2. Characterization

Inorganic anions and cations were analyzed using an ion chromatograph spectrometer (Thermo ICS 2000, USA) and an inductively coupled plasma spectrometer (ICP, Perkin Elmer Optima 7000DV, USA), respectively. Before each ICP and ICS test, a microwave digestion device (Analytik TOP wave, Germany) was employed to digest the organic components that could cause possible interference. For organic components, COD values of the feed and permeate solution were measured using a spectrophotometer (Hach DR 3900, USA). The values of conductivity and total dissolved solids were both measured by a conductivity meter (Mettler Toledo FE 38, China). Specific organic components (including esters, alcohols, benzenes, and others) were tested by a triple-quadrupole mass analyzer (Bruker Scion-MS-4306GC, Germany). An atomic force microscope (AFM) (Bruke Icon, Germany) operated in tapping mode was used to evaluate the surface topography of the membranes in a scan size of 10 × 10 μm.

Since the original aniline wastewater had undergone many complex treatment procedures in the industry, it was important to determine its composition and properties. The basic information on the aniline wastewater is shown in Table 1. The untreated aniline wastewater was alkaline, the cations were mainly sodium ions, with a content of about 52.86 g·L^−1^, the anions were mainly chloride ions, with a content of about 66.30 g·L^−1^, and trace nitrate ions were also present in the aniline wastewater. In addition, the conductivity value was about 162.75 ms·cm^−1^, and the COD and TOC (total dissolved solids) values were about 1400 mg·L^−1^ and 4728 mg·L^−1^, respectively. Based on this information, it was speculated that the aniline wastewater mainly contained NaCl. In addition, the specific organic components are listed in Table S1 and Figures S1 and S2. Seven kinds of main organics were detected in the aniline wastewater, among which aniline was the main pollutant.

In order to evaluate the effects of aniline wastewater on the membranes before and after MD operation, the membrane morphology and surface element distribution were measured using a field emission scanning electron microscope–energy dispersive spectrometer (FESEM-EDS, Hitachi S4800, Japan). The average pore size and pore size distribution of the membranes were measured by a membrane pore size distribution apparatus (PSDA-20, Gaoqian function Co., Nanjing, China) based on the gas–liquid exclusion method.

The porosity of the PVDF membranes (ε) was measured by the gravimetric method and calculated by Equation (1):

(1) $$\varepsilon \left(\%\right)=\frac{\frac{W_{w}-W_{d}}{\rho_{i}}}{\frac{W_{w}-W_{d}}{\rho_{i}}+\frac{W_{d}}{\rho_{M}}}\times 100\%$$

where W_w_ is the weight of the wet membrane, W_d_ is the weight of the dry membrane, $\rho_{i}$ is the density of IPA, and $\rho_{M}$ is the density of PVDF. Mechanical strength tests were performed with a tensile strength testing instrument (Model SH-20, Wenzhou Shandu Instrument Co., China). Then, tensile strength (σ) and elongation at break (δ) were calculated by Equations (2) and (3), respectively:

(2) $$\sigma =\frac{F}{A}\times 100\%$$

(3) $$\delta =\frac{L-L_{0}}{L_{0}}\times 100\%$$

where A is the cross-sectional area of the membrane sample, L and F are the final length and tensile stress when the sample was broken, and L_0_ is the initial length of the tested sample. Hydrophobicity is one of the most significant parameters of the membranes used in MD and is usually evaluated by contact angle (CA) measurements. In the present work, CA was measured by a commercial contact angle instrument (Dataphysics OCA 25, Germany).

### 2.3. Fenton Oxidation Pretreatment Process

The Fenton oxidation process is one of the processes of the catalytic oxidation of organic substances in wastewater by hydroxyl radicals generated by the reaction of ferrous ions (Fe^2+^) and H_2_O_2_. Compared with other methods, the Fenton oxidation process has the advantages of economy, convenient use, non-toxic by-product production, and no need for any complex or expensive instruments [41,42]. Ferrous ions catalyze hydrogen peroxide to produce hydroxyl radicals as follows:

Fe^2+^ + H_2_O_2_ → Fe^3+^ +·OH + OH^−^

During the Fenton reaction, iron ions and hydroxyl ions are also formed due to the reaction of oxygen free radicals and ferrous ions.

Fe^3+^ + H_2_O_2_ → Fe^2+^ + H^+^ +HO_2_

OH + Fe^2+^ → OH^−^ + Fe^3+^

OH + H_2_O_2_ → H_2_O + HO_2_

OH +·OH → H_2_O_2_

The removal rate of aniline wastewater can be calculated by Equation (4) as follows:

(4) $$\;\mathrm{Aniline}\;\mathrm{removal}\;\mathrm{rate}=\frac{C_{0}-L_{0}}{C_{0}}\times 100\%$$

where C_1_ and C_0_ are the concentration detected by an ultraviolet spectrophotometer (Perkin Elmer Lambda 950, USA) of original aniline wastewater and aniline wastewater after pretreatment [43,44].

### 2.4. Vacuum Membrane Distillation and Crystallization

The membrane used in this experiment was a Millipore GVHP 0.22 μm hydrophobic membrane, and its specific parameters are shown in Table 2.

**Figure 1:**
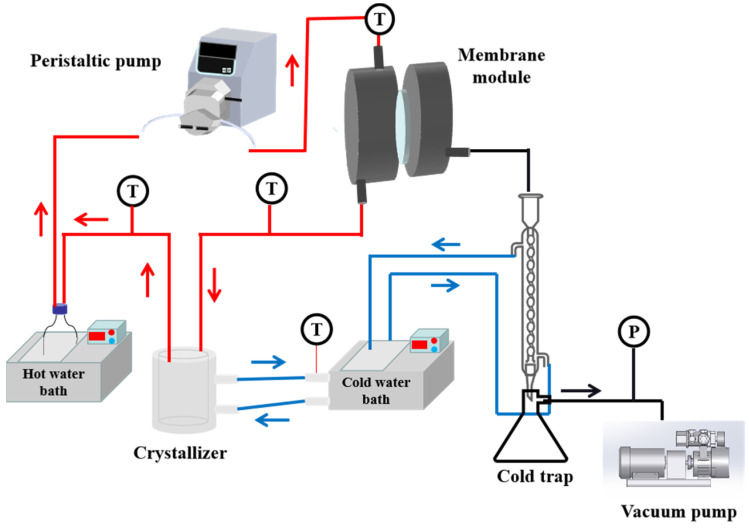
The experimental setup of membrane distillation crystallization (the red lines represent the hot flow and the blue lines represent the cold flow).

The performance of aniline wastewater treatment was tested by using the experimental MDCr setup, as shown in Figure 1. The effective membrane area was 11.34 cm^2^, and the permeate vacuum degree of permeate side was kept around 95 kPa. In addition, in order to promote crystallization, stirring was performed in the crystallizer. A total volume of 1 L aniline wastewater was circulated by a peristaltic pump. On the permeate side, the cooling bath temperature was kept at 5 °C. For each test, the permeate and conductivity were recorded. The permeate flux (*J*) and concentration factor of the feed solution were calculated by Equations (5) and (6) [45].

(5) $$\;J=\frac{m}{A\times t}$$

(6) $$\;\mathrm{Concentration}\;\mathrm{factor}=\frac{Q_{0}}{Q_{0}-Q_{p}}$$

where m is the mass of the permeable liquid (kg), A is the effective area of the flat membrane (m^2^), t is the time interval (h) of operation, and $Q_{0}$ and $Q_{p}$ are the initial quantity of the feed and the cumulative quantity of permeated water, respectively.

A conductivity meter was used to measure the conductivity of the product water, and the salt rejection can be calculated by Equation (7) as follows:

(7) $$\;\mathrm{Salt}\;\mathrm{rejection}=\frac{C_{f}-C_{p}}{C_{f}}\times 100\%$$

where $C_{f}$ and $C_{p}$ are the conductivity of the feed solution and the permeate solution, respectively.

## 3. Results and Discussion

### 3.1. Optimization of Operating Parameters for VMD Treatment of Aniline Wastewater

**Figure 2:**
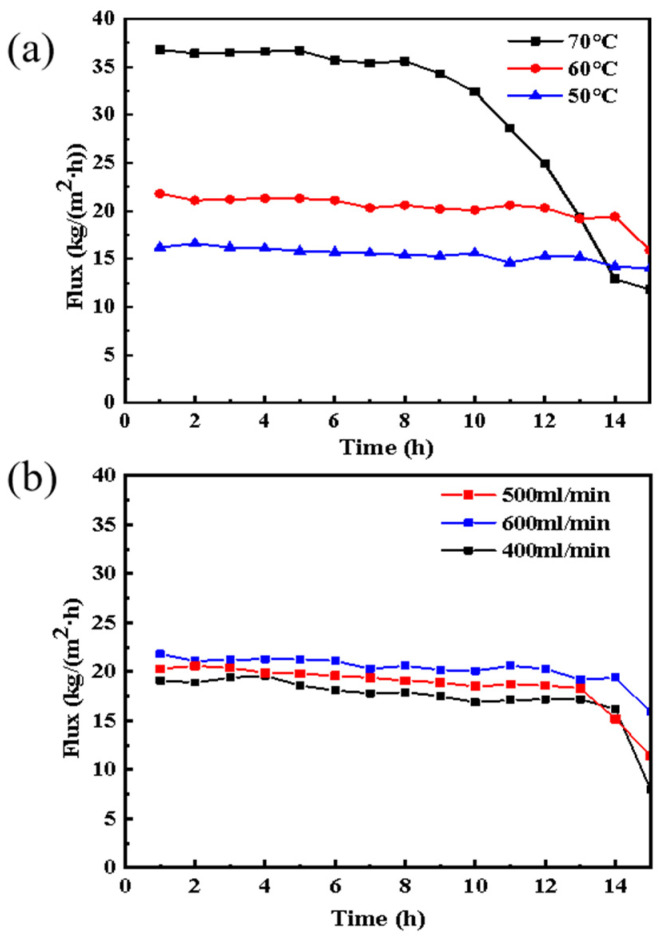
VMD flux of treating aniline wastewater under different feed temperatures (**a**) and flow rates (**b**).

Under the conditions of different feed temperatures of 50 °C, 60 °C, and 70 °C, feed flow of 500 mL/L, and absolute pressure of 5 kPa at the permeate side, the experiment of treating aniline wastewater with VMD for 15 h was carried out. Figure 2a shows the MD flux variation with different feed temperatures. Among them, when the temperature was 50 °C, 60 °C, or 70 °C, the permeation flux of membrane distillation was 16.7, 21.8, and 36.9 kg·m^−2^·h^−1^, respectively. With the gradual increase in feed temperature, the saturated vapor pressure of the volatile components gradually increased at the corresponding temperature, which increased the mass transfer impetus and permeation flux of the process [46,47]. At 50 °C and 60 °C, the permeation flux of VMD decreases slightly but is relatively stable within 15 h, however, when the feed temperature was 70 °C, the initial flux was relatively higher. After 8 h of operation, the flux began to decay, and after 14 h of operation, the flux dropped from 36.9 kg·m^−2^·h^−1^ at the beginning to about 14.5, a 50% drop in flux. This probably occurred because the more volatile substances clogged the pores along with the water steam when going through the membrane pore channel at higher temperatures; pollutant deposition also occurred on the membrane surface and so the pollution layer was thickened [48,49].

An experiment was carried out to evaluate the influence of different feed flow rates of 400, 500, and 600 mL/min under the conditions of a feed temperature of 60 °C and absolute pressure of 5 kPa at the permeate side. The results are shown in Figure 2b. The initial permeation flux was 18.8, 21.3, and 22.3 kg·m^−2^·h^−1^, corresponding to different flow rates of 400, 500, and 600 mL/min, respectively. With the increase in the feed flow rate, the permeation flux of MD gradually increased. The high flow rate is conducive to the turbulent state, which enables better mixing of the feed solution and decreases the thermal boundary layer and temperature polarization [50,51]. However, the increasing trend is not obvious relative to different temperatures because the temperature is the main influence factor of membrane distillation flux [52,53].

**Figure 3:**
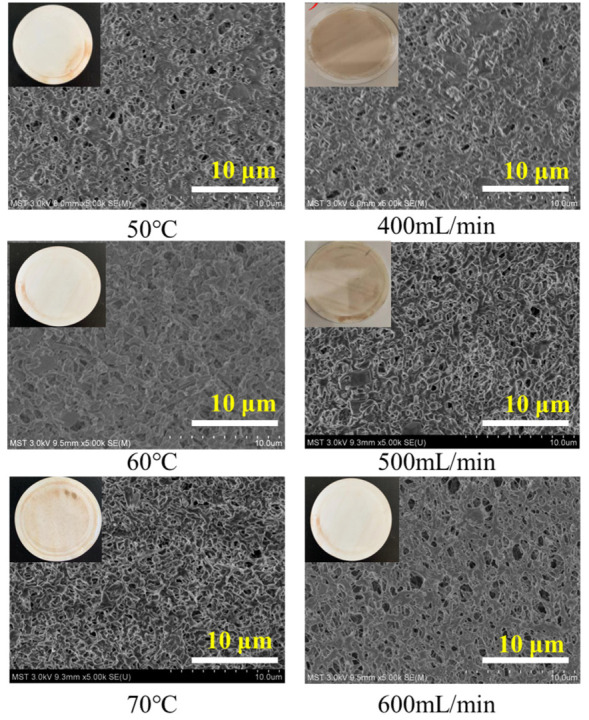
SEM images after 15 h VMD at different temperatures (**left**) and flow rates (**right**).

In addition to studying the flux changes under different MD operating conditions, we also investigated the fouling of the membrane surface after the vacuum membrane distillation treatment of aniline wastewater, as shown in Figure 3. As seen in the SEM images, with the increase in feed temperature, the fouling phenomenon on the membrane surface became more serious; the color changes can be seen in the pictures in the upper left corners of the SEM images. Correspondingly, it can be observed from SEM that more and more pollutants accumulated on the membrane surface, resulting in fewer and fewer visible pores. This was attributed to the complex organic composition of aniline wastewater. More and more foulants accumulated on the membrane’s surface or volatilized into the pores, which led to a rapid decay of membrane distillation flux after a few hours at 70 °C [54]. However, the influence of the flow rate on membrane surface fouling presented a completely opposite trend. It can be seen from the SEM images and photos of the membrane surface that the membrane surface fouling was more serious when the flow rate was 400 mL/min, while the fouling was not serious when the flow rate was 600 mL/min. This is because an increase in the flow rate will increase the Reynolds number, and a high flow rate is conducive to obtaining a turbulent flow state. A turbulent state can better relieve the deposition of fouling on the membrane surface [55,56].

As is shown in Table 3, when the temperature was 60 °C and the flow rate was 400, 500, and 600 mL/min, the average surface roughness of the membrane decreased by 50.7%, 17.9%, and 15.9%, respectively. This is because when the flow velocity increases, the concentration of pollutants in the feed liquid accumulates on the surface of the membrane during a long membrane distillation; the deposition of organic matter also resulted in a decrease in surface hydrophobicity [57], which also explains the lower average surface roughness at the flow rate of 400 mL/min. This result is consistent with the SEM results. Under the flow rate condition of 500 mL/min and temperatures of 50, 60, and 70 °C, the average surface roughness of the membrane decreased by 56.1%, 20.7%, and 86.5%, respectively. This is because when the temperature is low, the diffusion coefficient of the organic matter in the wastewater is low, and the membrane surface pollution deposition is fast, so the average surface roughness is low. When the temperature was too high, the aniline wastewater was concentrated quickly, the pollutant concentration in the wastewater increased, and a large number of pollutants were deposited on the membrane surface, which made the membrane surface smooth, decreased the hydrophobicity, and finally, wetted the membrane [58,59]. Therefore, using an appropriate temperature was very important for the MD process.

### 3.2. Pretreatment of Aniline Wastewater by Fenton Reaction

**Figure 4:**
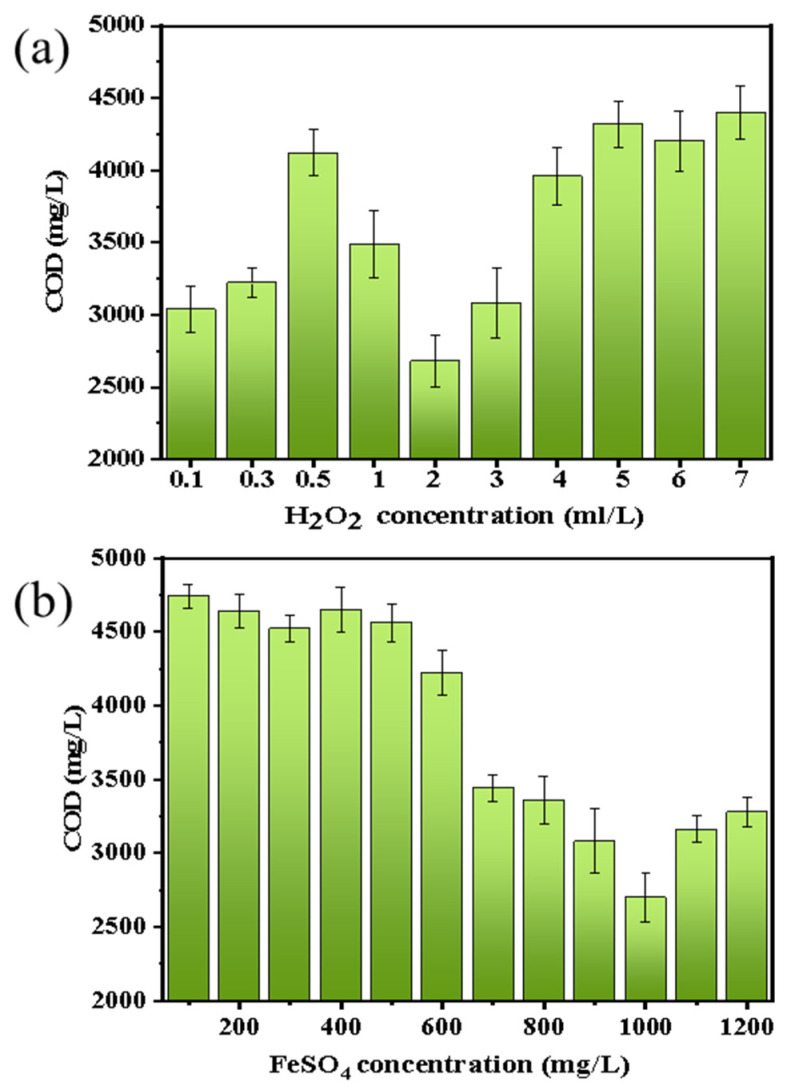
The effect of the concentration of (**a**) H_2_O_2_ and (**b**) FeSO_4_ in Fenton oxidation on the COD of aniline wastewater.

**Figure 5:**
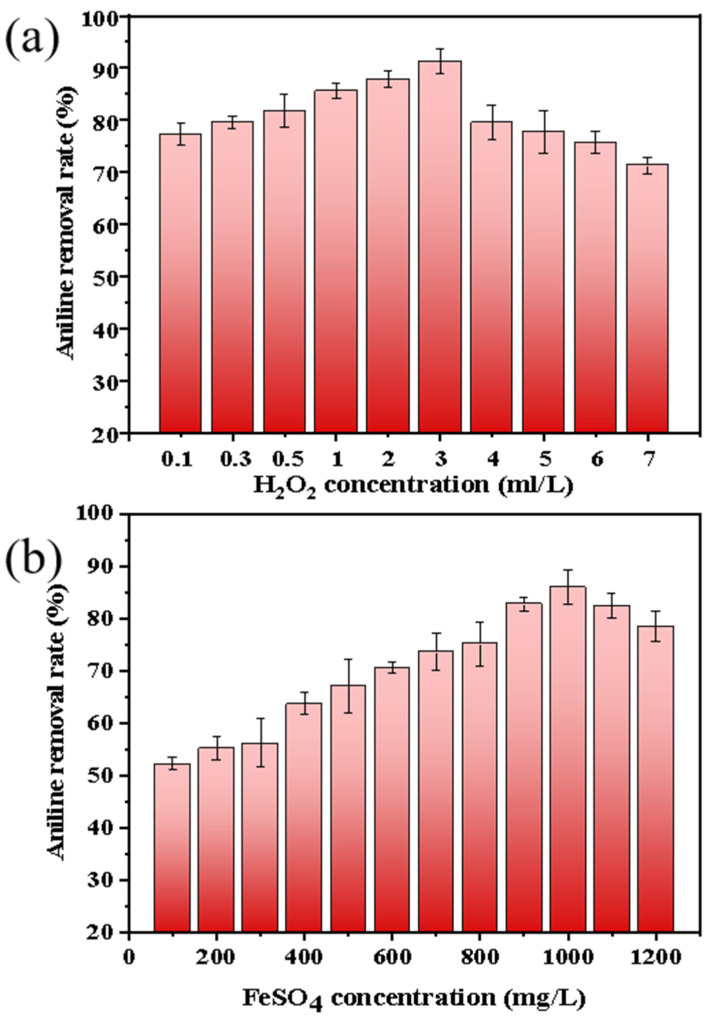
The effect of the concentration of (**a**) H_2_O_2_ and (**b**) FeSO_4_ on the aniline removal rate in Fenton oxidation.

**Figure 6:**
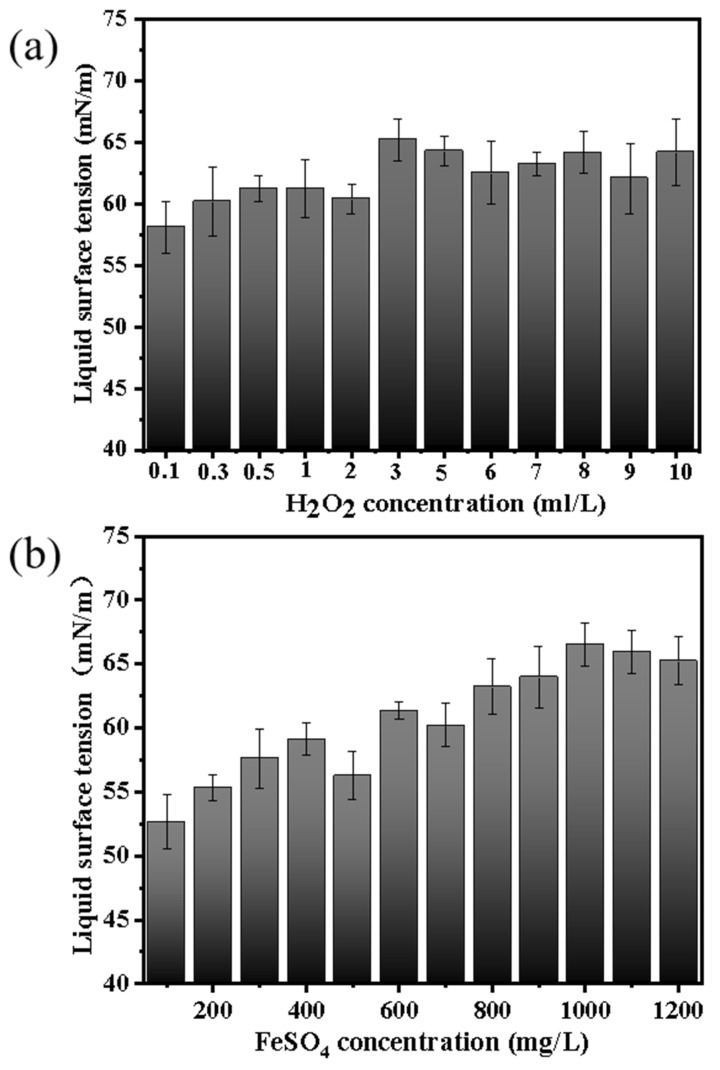
The effect of the concentration of (**a**) H_2_O_2_ and (**b**) FeSO_4_ in Fenton oxidation on the liquid surface tension.

**Figure 7:**
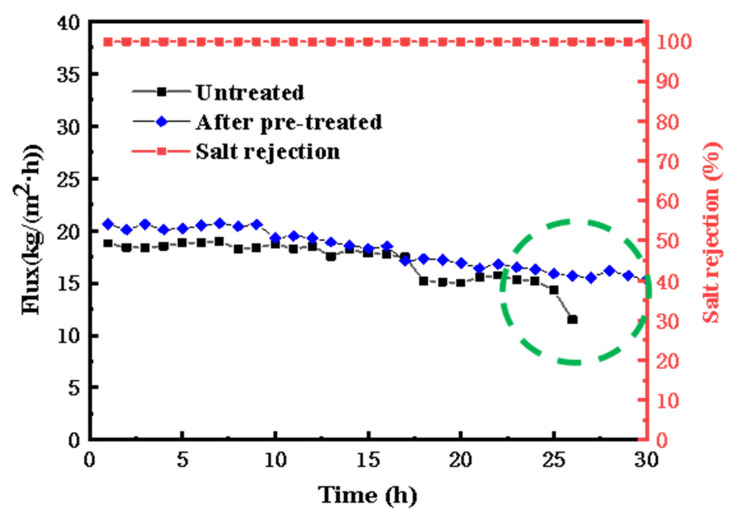
The VMD flux and salt rejection of untreated aniline wastewater and aniline wastewater after being pretreated.

Since the organic foulants on the membrane surface cannot be effectively treated during the membrane cleaning process, it was planned to use the Fenton oxidation method to pretreat the aniline wastewater to remove the aniline content in the aniline wastewater. Figure 4, Figure 5, Figure 6 and Figure 7 show the change of COD, aniline removal rate, and liquid surface tension under different H_2_O_2_ and FeSO_4_ concentrations, respectively.

Firstly, when the concentration of H_2_O_2_ was 0.1, 0.3, 0.5, 1, 2, 3, 4, 5, 6, and 7 mL/L (FeSO_4_ was 1000 mg/L), the COD, aniline removal rate, and liquid surface tension in the wastewater were measured; the results are shown in Figure 4a, Figure 5a, Figure 6a. The COD value shows a trend of first decreasing and then increasing with the increase in H_2_O_2_ concentration. When the H_2_O_2_ concentration was 3 mL/L, the COD reached its lowest value. Due to the increase in the amount of H_2_O_2_ added to the Fenton reaction, the output of ·OH increased, and the removal rate of the organic foulants also increased. However, when excessive H_2_O_2_ was added, it inhibited the generation of ·OH, and H_2_O_2_ itself constantly decomposed into oxygen and water, oxidizing Fe^2+^ into Fe^3+^ with low catalytic ability, increasing the amount of hydrogen peroxide, and reducing the production efficiency of the ·OH radical [60,61,62,63,64]. The change of COD also partly explains why the removal rate of aniline increases first and then decreases.

Especially, at the concentration of 3 mL/L H_2_O_2_ and 1000 mg/mL FeSO_4_, the liquid surface tension of the aniline wastewater increased, and the liquid surface tension increased, albeit by a small amount. This is a good performance for MD. Because ·OH has strong oxidation, it will oxidize and decompose aniline, thus increasing the liquid surface tension of wastewater. According to Laplace’s equation, an increase in liquid surface tension will reduce the occurrence of membrane wetting during membrane distillation.

When wetting, the liquid enters the membrane hole, as seen in the Laplace Equation (8):

(8) $$\Delta P=\frac{2\gamma_{1}\cos\theta }{r}$$

where ΔP is the pressure difference on the membrane surface, $\gamma_{1}$ is the liquid surface tension, r is the pore size of the membrane, and θ is the contact angle of the membrane surface. When θ>90°, cosθ<0, ΔP>0, and only when a certain pressure is applied, will the liquid penetrate into the membrane pores. From the equation, it can be seen that wettability depends on three factors: membrane pore size, liquid surface tension, and membrane material surface energy. The smaller the liquid surface tension, the easier it is to cause membrane wetting.

Secondly, the results from a FeSO_4_ concentration of 100, 200, 300, 400, 500, 600, 700, 800, 900, 1000, 1100, and 1200 mg/L and an H_2_O_2_ concentration of 3 mL/L were also measured and are shown in Figure 4b, Figure 5b, Figure 6b. From Figure 4b, it can be seen that the COD removal rate increases with the increase in FeSO_4_ concentration. When the concentration is 1000 mg/L, the COD removal effect is the best. Afterward, the COD removal rate decreases with an increase in FeSO_4_ concentration. That is because when the mass concentration of Fe^2+^ is low, the amount of Fe^2+^ added increases, the catalytic effect is enhanced, the amount of ·OH free radicals generated increases, and the COD removal rate increases. When the mass concentration of Fe^2+^ is too high, it will reduce a portion of H_2_O_2_, causing the decomposition rate of H_2_O_2_ to be too fast and producing a large amount of ·OH in a short period of time. Thus, some ·OH cannot react with the organic pollutants in time, and free radicals will have already formed mutual reactions and been consumed, resulting in a decrease in the utilization rate of ·OH [65,66].

Figure 7 shows that the initial permeation flux of treating original aniline wastewater is about 18 kg·m^−2^·h^−1^ and the initial permeation flux after pretreatment is about 22 kg·m^−2^·h^−1^; the wastewater after pretreatment shows a more stable and lasting permeation flux in long-term operation.

### 3.3. Membrane Distillation and Crystallization Process

The performance of the hydrophobic MDCr process on Fenton oxidation-pretreated aniline wastewater will be discussed in the following section. The hydrophobic PVDF membrane was adopted during the process, and the experimental operation parameters of the MDCr process are shown in Table 4.

**Figure 8:**
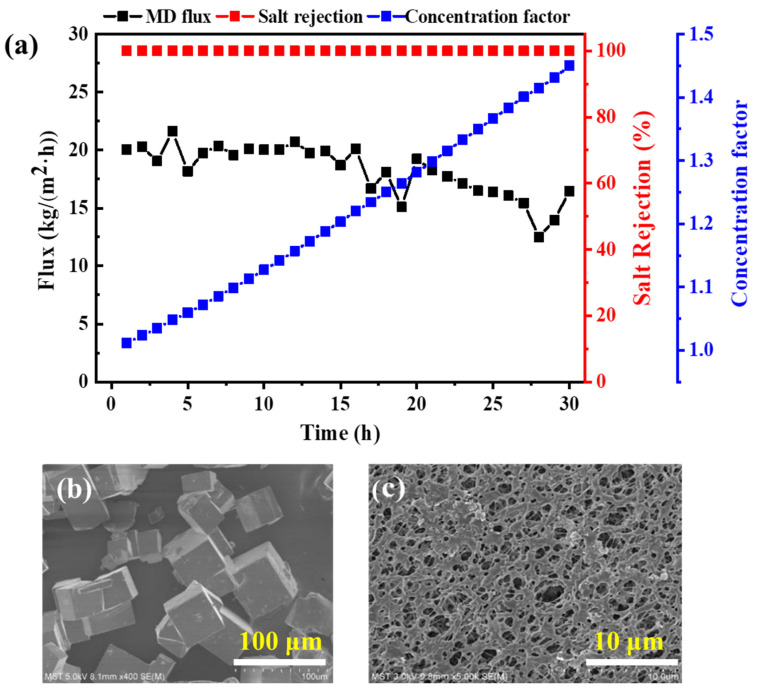
(**a**) MDCr treatment of aniline wastewater using a commercial PVDF membrane. (**b**) The EDX image of salt crystals on the membrane surface after MDCr operation and (**c**) the SEM image of the membrane surface after MDCr operation.

It can be seen from Figure 8a that during the 30 h operation process, the overall flux remains stable and the salt retention rate of the membrane remains relatively stable; both are above 99.99%. In addition, after 30 h, the concentration factor reached 1.5, which means that during this process, the original aniline wastewater had been concentrated to 1.5 times the original concentration. It can be seen from the SEM images (Figure 8b,c) of the membrane after the experiment that there was no obvious membrane pore blockage or large-scale pollutant deposition on the membrane surface, and that it still maintained a porous state.

The organic components in the original solution, concentrated crystallization mother solution, and crystallization salt were characterized, as shown in Table 5. It can be seen that the TOC value of the concentrated crystallization mother solution decreased compared to the original solution, and it has not been ruled out that the organic substances with lower boiling points can be removed due to their permeation through the membrane in a vacuum environment.

Crystalline salt precipitated during the membrane distillation crystallization process and was obtained through filtration and drying. In order to understand the residual organic compounds on the surface of the crystal salt, the crystal salt was reintegrated into deionized water (with a concentration of 10 ppm), and the TOC value of the crystal salt was measured to be 2.99 mg/L.

**Figure 9:**
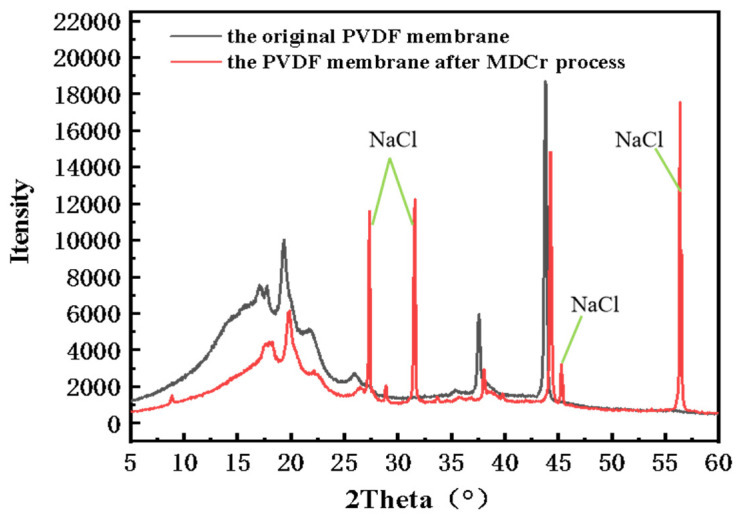
The XRD spectrum of the original PVDF membrane and the PVDF membrane after the MDCr process.

An EDX analysis was then performed on the crystalline salt, as shown in Figure 8b and Table 6. Figure 8b shows the surface morphology of the crystalline salt, a cubic shape with clear edges and a complete structure [32]. The salt surface analysis by EDX is shown in Table 5. The main elements are Na and Cl, and their atomic ratios are close to 1:1, which means the membrane distillation and crystallization process can be used to achieve ZLD of the aniline wastewater in this case. In order to further confirm this conclusion, we also conducted XRD characterization, as shown in Figure 9. The XRD spectrum of the PVDF membrane after the MDCr process showed obvious characteristic peaks of NaCl with peaks of 2θ = 27.36°, 31.73°, 45.44°, and 56.47°. This further confirmed that the crystals we obtained were NaCl. However, due to the complex composition of the wastewater, there were still some peaks of unknown organic and inorganic substances.

Nevertheless, when membrane distillation was used to treat aniline wastewater, although the Fenton oxidation process was used to pretreat the wastewater, the final membrane surface fouling was still very serious, and further modification of the membrane surface is needed to improve the antifouling performance. Moreover, the aniline wastewater obtained from the actual factory has complex components, so better pretreatment methods should be tried to further improve the long-term stability of membrane distillation.

## 4. Conclusions

In this paper, MDCr was applied to the treatment of aniline wastewater, providing a feasible solution for the realization of ZLD. When the feed temperature is 60 °C and the feed flow rate is 500 mL/min, the flux of the MD process is 21.3 kg·m^−2^·h^−1^; under this condition, the effect of MD is the best and the influence of foulants on the performance of MD can be avoided to the greatest extent. After that, the concentration of H_2_O_2_ and FeSO_4_ in Fenton oxidation was optimized, and when the optimal concentration of H_2_O_2_, 3 mL/L, and FeSO_4_, 1000 mg/L, was discovered, the aniline removal rate was found to reach 91%. Finally, the salt was extracted and analyzed. The purity of the NaCl was up to 94 wt.%. Overall, in this case, the MDCr process can be used to achieve ZLD of the aniline wastewater, showing the possibility and great potential of the large-scale treatment of aniline wastewater.

## Figures and Tables

**Table 1:** Basic information on aniline wastewater.

Number	Project	Units	Numerical Value
1	pH	-	12.85–12.96
2	Conductivity	ms·cm^−1^	162.75
3	Na^+^	g·L^−1^	52.86
4	Cl^-^	g·L^−1^	66.30
5	NO^3-^	g·L^−1^	0.88
6	Turbidity	NTU	2.98
7	TDS	mg·L^−1^	≈125
8	TOC	mg·L^−1^	4728
9	COD	mg·L^−1^	5600

**Table 2:** Basic parameters of the PVDF membrane.

PVDF Characteristics	Value
Contact angle (°)	128
Porosity (%)	65.12
Tensile ratio (%)	69.05
Tensile strength (MPa)	4.52
Pore size (nm)	334
Thickness (mm)	0.122

**Table 3:** AFM images of the original PVDF membrane and the PVDF membrane after operation in different conditions.

Operating Condition	Mean Roughness (R_a_), nm	AFM Images
Original Membrane	207	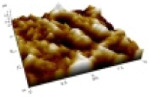
60 °C/400 mL/min	102	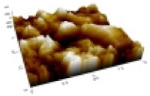
60 °C/500 mL/min	170	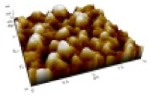
60 °C/600 mL/min	174	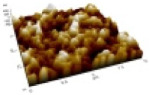
500 mL/min/50 °C	90.8	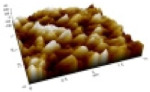
500 mL/min/60 °C	164	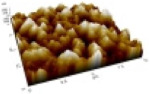
500 mL/min/70 °C	28	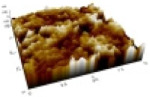

**Table 4:** The operation parameters of membrane distillation and crystallization.

Processing Capacity/L	Feed Flow Rate /mL∙min^−1^	Feed Temperature /°C	Vacuum Pressure /MPa
2	500	60	0.0095

**Table 5:** Analysis of the organic composition of wastewater and crystal salt.

TOC (mg/L)		
Original Solution	Crystalline Mother Solution	Crystalline Salt (10 ppm)
4728	2471.1	2.99

**Table 6:** Surface element analysis of the crystal salt.

Element	Mass Ratio %	Atomic Ratio %
O	4.54	7.95
Na	38.66	47.14
Cl	56.80	44.91

## Data Availability

The data presented in this study are available on request from the corresponding author.

## Supplementary Materials

The following supporting information can be downloaded at: https://www.mdpi.com/article/10.3390/membranes13060561/s1, Table S1: Organic content in aniline wastewater; Figure S1: GC-MS spectrum of aniline wastewater; Figure S2: The pore size distribution of the original membrane and the fouled membrane.

## References

[1] Nie Y., Deng Z., Yuan J. (2003). Application and development of treatment technology for aniline wastewater. Tech. Equip. Environ. Pollut. Control.

[2] Zhang Q., Zhang W., He Q., Li M., Li Y., Huang W. (2020). Effects of dissolved oxygen concentrations on a bioaugmented sequencing batch rector treating aniline-laden wastewater: Reactor performance, microbial dynamics and functional genes. Bioresour. Technol..

[3] Hussain I., Zhang Y., Li M., Huang S., Hayat W., He L., Du X., Liu G., Du M. (2018). Heterogeneously degradation of aniline in aqueous solution using persulfate catalyzed by magnetic BiFeO_3_ nanoparticles. Catal. Today.

[4] Yang K., Ji M., Liang B., Zhao Y., Zhai S., Ma Z., Yang Z. (2020). Bioelectrochemical degradation of monoaromatic compounds: Current advances and challenges. J. Hazard. Mater..

[5] Zhou L., Yan X., Yan Y., Li T., An J., Liao C., Li N., Wang X. (2020). Electrode potential regulates phenol degradation pathways in oxygen-diffused microbial electrochemical system. Chem. Eng. J..

[6] Zhou M., Wang W., Chi M. (2009). Enhancement on the simultaneous removal of nitrate and organic pollutants from groundwater by a three-dimensional bio-electrochemical reactor. Bioresour. Technol..

[7] Jiang Y., Shang Y., Gong T., Hu Z., Yang K., Shao S. (2020). High concentration of Mn2+ has multiple influences on aerobic granular sludge for aniline wastewater treatment. Chemosphere.

[8] Devulapalli R., Jones F. (1999). Separation of aniline from aqueous solutions using emulsion liquid membranes. J. Hazard. Mater..

[9] Liu Y., Shen X., Sun J., Li P., Zhang A. (2019). Treatment of aniline contaminated water by a self-designed dielectric barrier discharge reactor coupling with micro-bubbles: Optimization of the system and effects of water matrix. J. Chem. Technol. Biotechnol..

[10] Yu S., Wang X., Chen Z., Wang J., Wang S., Hayat T., Wang X. (2017). Layered double hydroxide intercalated with aromatic acid anions for the efficient capture of aniline from aqueous solution. J. Hazard. Mater..

[11] Figoli A., Ursino C., Galiano F., Di Nicolò E., Campanelli P., Carnevale M., Criscuoli A. (2017). Innovative hydrophobic coating of perfluoropolyether (PFPE) on commercial hydrophilic membranes for DCMD application. J. Membr. Sci..

[12] Iqhrammullah M., Fahrina A., Chiari W., Ahmad K., Fitriani F., Suriaini N., Safitri E., Puspita K. (2023). Laccase Immobilization Using Polymeric Supports for Wastewater Treatment: A Critical Review. Macromol. Chem. Phys..

[13] Mei Y., Tang C.Y. (2018). Recent developments and future perspectives of reverse electrodialysis technology: A review. Desalination.

[14] Deshmukh A., Boo C., Karanikola V., Lin S., Straub A.P., Tong T., Warsinger D.M., Elimelech M. (2018). Membrane distillation at the water-energy nexus: Limits, opportunities, and challenges. Energy Environ. Sci..

[15] Reddy A.S., Kalla S., Murthy Z. (2022). Textile wastewater treatment via membrane distillation. Environ. Eng. Res..

[16] Shao Y., Han M., Wang Y., Li G., Xiao W., Li X., Wu X., Ruan X., Yan X., He G. (2019). Superhydrophobic polypropylene membrane with fabricated antifouling interface for vacuum membrane distillation treating high concentration sodium/magnesium saline water. J. Membr. Sci..

[17] Zhu Z., Zhong L., Horseman T., Liu Z., Zeng G., Li Z., Lin S., Wang W. (2021). Superhydrophobic-omniphobic membrane with anti-deformable pores for membrane distillation with excellent wetting resistance. J. Membr. Sci..

[18] Shao S., Shi D., Hu J., Qing W., Li X., Li X., Ji B., Yang Z., Guo H., Tang C.Y. (2022). Unraveling the Kinetics and Mechanism of Surfactant-Induced Wetting in Membrane Distillation: An In Situ Observation with Optical Coherence Tomography. Environ. Sci. Technol..

[19] García-Payo M.C., Essalhi M., Khayet M. (2010). Effects of PVDF-HFP concentration on membrane distillation performance and structural morphology of hollow fiber membranes. J. Membr. Sci..

[20] Hou D., Wang J., Sun X., Ji Z., Luan Z. (2012). Preparation and properties of PVDF composite hollow fiber membranes for desalination through direct contact membrane distillation. J. Membr. Sci..

[21] Feng C., Khulbe K.C., Matsuura T., Gopal R., Kaur S., Ramakrishna S., Khayet M. (2008). Production of drinking water from saline water by air-gap membrane distillation using polyvinylidene fluoride nanofiber membrane. J. Membr. Sci..

[22] Singh D., Sirkar K.K. (2012). Desalination by air gap membrane distillation using a two hollow-fiber-set membrane module. J. Membr. Sci..

[23] Lee C.H., Hong W.H. (2001). Effect of operating variables on the flux and selectivity in sweep gas membrane distillation for dilute aqueous isopropanol. J. Membr. Sci..

[24] Khayet M., Godino M., Mengual J. (2003). Theoretical and experimental studies on desalination using the sweeping gas membrane distillation method. Desalination.

[25] Ji H., Choi M.-Y., Lee H.-S., Kim A.S., Kim H.-J. (2017). Vacuum membrane distillation for deep seawater: Experiments and theory. Desalination Water Treat..

[26] Mengual J., Khayet M., Godino M. (2004). Heat and mass transfer in vacuum membrane distillation. Int. J. Heat Mass Transf..

[27] Mericq J.-P., Laborie S., Cabassud C. (2009). Vacuum membrane distillation for an integrated seawater desalination process. Desalination Water Treat..

[28] Banat F.A., Simandl J. (1999). Membrane distillation for dilute ethanol: Separation from aqueous streams. J. Membr. Sci..

[29] Cerneaux S., Strużyńska I., Kujawski W.M., Persin M., Larbot A. (2009). Comparison of various membrane distillation methods for desalination using hydrophobic ceramic membranes. J. Membr. Sci..

[30] Banat F., Jwaied N. (2008). Economic evaluation of desalination by small-scale autonomous solar-powered membrane distillation units. Desalination.

[31] Ben Abdallah S., Frikha N., Gabsi S. (2013). Simulation of solar vacuum membrane distillation unit. Desalination.

[32] Curcio E., Drioli E. (2005). Membrane Distillation and Related Operations—A Review. Sep. Purif. Rev..

[33] Moradi R., Monfared S.M., Amini Y., Dastbaz A. (2016). Vacuum enhanced membrane distillation for trace contaminant removal of heavy metals from water by electrospun PVDF/TiO_2_ hybrid membranes. Korean J. Chem. Eng..

[34] Shi W., Li T., Tian Y., Li H., Fan M., Zhang H., Qin X. (2022). An innovative hollow fiber vacuum membrane distillation-crystallization (VMDC) coupling process for dye house effluent separation to reclaim fresh water and salts. J. Clean. Prod..

[35] Mene N.R., Murthy Z. (2019). Recovery of pure water and crystalline products from concentrated brine by using membrane distillation crystallization. Sep. Sci. Technol..

[36] Park S.H., Kim J.H., Moon S.J., Jung J.T., Wang H.H., Ali A., Quist-Jensen C.A., Macedonio F., Drioli E., Lee Y.M. (2020). Lithium recovery from artificial brine using energy-efficient membrane distillation and nanofiltration. J. Membr. Sci..

[37] Pan J., Chen M., Xu X., Sun S.-P., Wang Z., Cui Z., Xing W., Tavajohi N. (2022). Enhanced anti-wetted PVDF membrane for pulping RO brine treatment by vacuum membrane distillation. Desalination.

[38] Di Profio G., Grosso V., Caridi A., Caliandro R., Guagliardi A., Chita G., Curcio E., Drioli E. (2011). Direct production of carbamazepine–saccharin cocrystals from water/ethanol solvent mixtures by membrane-based crystallization technology. CrystEngComm.

[39] Tong T., Elimelech M. (2016). The Global Rise of Zero Liquid Discharge for Wastewater Management: Drivers, Technologies, and Future Directions. Environ. Sci. Technol..

[40] Elimelech M., Phillip W.A. (2011). The Future of Seawater Desalination: Energy, Technology, and the Environment. Science.

[41] Benredjem Z., Barbari K., Chaabna I., Saaidia S., Djemel A., Delimi R., Douas S., Bakhouche K. (2021). Comparative investigation on the removal of methyl orange from aqueous solution using three different advanced oxidation processes. Int. J. Chem. React. Eng..

[42] Luo J., Tang Y., Zhou J., Yang Y. (2014). Experimental study on the treatment process of wastewater in a printing and dyeing industrial park. Ind. Water Treat..

[43] Gong Y., Xu X., Wang F., Zhao L., Chen Y., Shen S. (2008). Study on the pretreatment of aniline wastewater by combining micro-electrolysis with Fenton oxidation. Ind. Water Treat..

[44] Zhao D., Zou H., Zhu Q., Yang X. (2012). Aniline wastewater treatment by Fenton oxidation-coagulation. Chin. J. Environ. Eng..

[45] Li X., Zhang Y., Cao J., Wang X., Cui Z., Zhou S., Li M., Drioli E., Wang Z., Zhao S. (2019). Enhanced fouling and wetting resistance of composite Hyflon AD/poly(vinylidene fluoride) membrane in vacuum membrane distillation. Sep. Purif. Technol..

[46] Ve Q.L., Koirala R., Bawahab M., Faqeha H., Do M.C., Nguyen Q.L., Date A., Akbarzadeh A. (2021). Experimental investigation of the effect of the spacer and operating conditions on mass transfer in direct contact membrane distillation. Desalination.

[47] Chen L., Xu P., Wang H. (2020). Interplay of the Factors Affecting Water Flux and Salt Rejection in Membrane Distillation: A State-of-the-Art Critical Review. Water.

[48] Olatunji S.O., Camacho L.M. (2018). Heat and Mass Transport in Modeling Membrane Distillation Configurations: A Review. Front. Energy Res..

[49] Moradi R., Karimi-Sabet J., Shariaty-Niassar M., Amini Y. (2016). Experimental investigation of nanofibrous poly(vinylidene fluoride) membranes for desalination through air gap membrane distillation process. Korean J. Chem. Eng..

[50] Nakoa K., Date A., Akbarzadeh A. (2015). A research on water desalination using membrane distillation. Desalination Water Treat..

[51] Rezaei M., Warsinger D.M., Duke M.C., Matsuura T., Samhaber W.M. (2018). Wetting phenomena in membrane distillation: Mechanisms, reversal, and prevention. Water Res..

[52] Zhang J., Dow N., Duke M., Ostarcevic E., Li J.-D., Gray S. (2010). Identification of material and physical features of membrane distillation membranes for high performance desalination. J. Membr. Sci..

[53] Abu-Zeid M.A.E.R., Zhang Y., Dong H., Zhang L., Chen H.L., Hou L. (2015). A comprehensive review of vacuum membrane distillation technique. Desalination.

[54] Stamatakis E., Stubos A., Palyvos J., Chatzichristos C., Muller J. (2005). An improved predictive correlation for the induction time of CaCO_3_ scale formation during flow in porous media. J. Colloid Interface Sci..

[55] Kim Y.-D., Francis L., Lee J.-G., Ham M.-G., Ghaffour N. (2018). Effect of non-woven net spacer on a direct contact membrane distillation performance: Experimental and theoretical studies. J. Membr. Sci..

[56] Abdallah H., Moustafa A., AlAnezi A.A., El-Sayed H. (2014). Performance of a newly developed titanium oxide nanotubes/polyethersulfone blend membrane for water desalination using vacuum membrane distillation. Desalination.

[57] Ricceri F., Blankert B., Ghaffour N., Vrouwenvelder J.S., Tiraferri A., Fortunato L. (2022). Unraveling the role of feed temperature and cross-flow velocity on organic fouling in membrane distillation using response surface methodology. Desalination.

[58] Charfi A., Tibi F., Kim J., Hur J., Cho J. (2021). Organic Fouling Impact in a Direct Contact Membrane Distillation System Treating Wastewater: Experimental Observations and Modeling Approach. Membranes.

[59] Naidu G., Jeong S., Kim S.-J., Kim I.S., Vigneswaran S. (2014). Organic fouling behavior in direct contact membrane distillation. Desalination.

[60] Guomin C.A.O., Ding W., Yang G., Zhang D. (2008). Aqueous Phase Degradation of Aromatic Compounds by Fenton Oxidation. J. East China Univ. Sci. Technoloy Nat. Sci. Ed..

[61] Zhang H., Zhou Y., Guo S., Lu X. (2021). Advances of advanced oxidation process to treat aniline wastewater. Ind. Water Treat..

[62] Chen D., Qian J., Jiang S. (2022). Performance of Fenton Three-phase Catalytic Oxidation Process for Advanced Treatment of Dyeing and Printing Wastewater. China Water Wastewater.

[63] Chen W., Wei Z. (2004). Study on Fenton oxidation-coagulation process for the treatment of printing and dyeing wastewater. Ind. Water Treat..

[64] Xue D., Li C., Zhang L., Liu D., Jiang H., Li X. (2014). Oxidation treatment of printing and dyeing wastewater by flocculation-Fenton reagent. Chin. J. Environ. Eng..

[65] Jianhui S., Shengpeng S., Huiliang W. (2006). Progress of the research on Fenton oxidation technology in the treatment of industrial wastewater containing refractory organic matter. Ind. Water Treat..

[66] Zhao G., Sun J., Zhang Y., Wang H., Wu J. (2021). Research progress of advanced oxidation technology in treatment of printing and dyeing wastewater. Appl. Chem. Ind..

