# Supplementary material for: Treatment of Aniline Wastewater by Membrane Distillation and Crystallization
Source: Membranes (Basel). 2023 May 30;13(6):561. doi: 10.3390/membranes13060561 (PMC10301228; doi:10.3390/membranes13060561)
Supplement: Supplementary file 1 [file membranes-13-00561-s001.zip › membranes-2400928-supplementary.pdf]

Supplementary information

Table S1. Organic content in aniline wastewater.

**Retention time/min**	**Chemical compound**	**Molecular formula**	**Boiling point/ °C**	**Molecular weight/Da**
6.175	aniline	C_6H_7N	184.4	93
7.344	2,4,6-trimethyldecane	$C_{13}H_{28}$	204.3	184
8.999	Cyclohexyldimethoxymethylsilane	$C_9H_{20}O_2Si$	201.2	188
10.444	2,6,10,14-tetramethylheptadecane	$C_{21}H_{44}$	356.9	296
10.672	N-pentadecane	$C_{15}H_{32}$	270.0	212
16.304	2,6,10,15-tetramethylheptadecane	$C_{21}H_{44}$	356.9	296
21.190	Triphenylphosphine oxide	$C_{18}H_{15}OP$	360.0	278

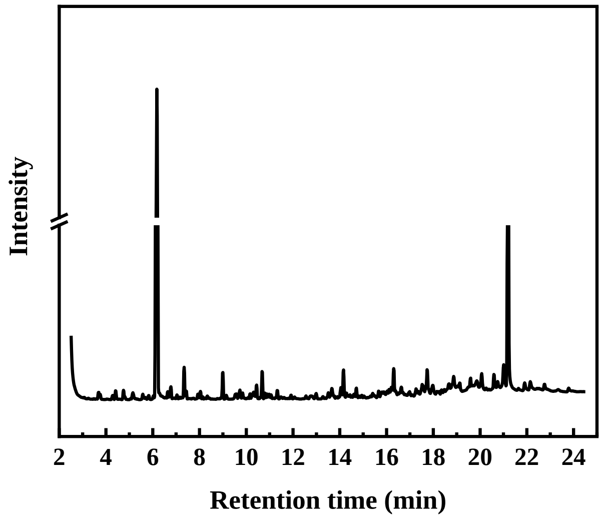

Figure S1 GC-MS spectrum of aniline wastewater.

As we can see, the pore size distribution of the fouled membrane at 0.25 um-0.30 um was reduced comparing the original membrane, this is because after the 15h MD operation, the membrane surface was polluted and some membrane pores were blocked, which can also explain that the flux in Figure 3 (b)began to decline at 15h.

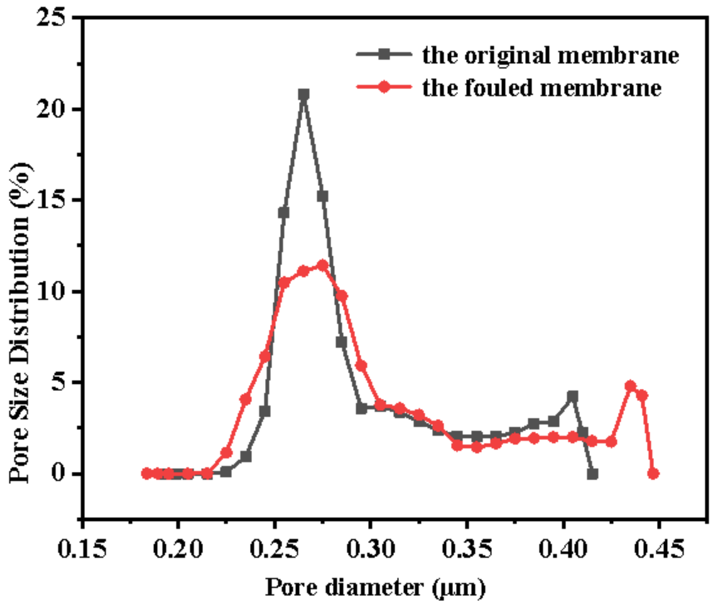

Figure S2. The pore size distribution of the original membrane and the fouled membrane.
